# Mechanical Enhancement of Sensitivity in Natural Rubber Using Electrolytic Polymerization Aided by a Magnetic Field and MCF for Application in Haptic Sensors

**DOI:** 10.3390/s16091521

**Published:** 2016-09-18

**Authors:** Kunio Shimada, Norihiko Saga

**Affiliations:** 1 Faculty of Symbiotic Systems Sciences, Fukushima University, 1 Kanayagawa, Fukushima 960-1296, Japan; 2 Department of Human System Interaction, Kansai Gakuin University, 2-1 Gakuen, Sanda, Hyogo 669-1337, Japan; saga@kwansei.ac.jp

**Keywords:** sensor, electrolytic polymerization, magnetic field, magnetic cluster, natural rubber, magnetic compound fluid (MCF), magnetic fluid, isoprene, filler, electrical resistivity

## Abstract

Sensors are essential to the fulfillment of every condition of haptic technology, and they need simultaneously to sense shear stress as well as normal force, and temperature. They also must have a strong and simple structure, softness, and large extension. To achieve these conditions simultaneously, we enhanced the sensitivity of sensors utilizing natural rubber (NR)-latex through the application of electrolytic polymerization focused on the isoprene C=C bonds in natural rubbers such as NR-latex, and then applied a magnetic field and magnetic compound fluid (MCF) as magnetically responsive fluid. When an electric field alone was used in the rubber, the effect of electrolytic polymerization was very small compared to the effect in well-known conductive polymer solution such as plastic. The MCF developed by Shimada in 2001 involved magnetite and metal particles, and acts as a filler in NR-latex. By utilizing the magnetic, electric fields and the MCF, we aligned the electrolytically polymerized C=C along the magnetic field line with the magnetic clusters formed by the aggregation of magnetite and metal particles so as to enhance the effect of electrolytic polymerization. We then demonstrated the effectiveness of the new method of rubber vulcanization on the sensitivity of the rubber by experimentally investigating its electric and dynamic characteristics.

## 1. Introduction

Sensor requirements include haptic sensibility, capability of sensing both shear stress and normal force for various sensing fields, sensing temperature, a strong and simple structure, softness, and large extension. Haptic sensors are commonly used in the field of robotics [1,2,3], and for sensing the surface roughness of various kinds of materials. Examples include tactile devices used in the machining of workpieces, sensing the roughness of human skin in the field of cosmetics, etc. In the former example, an apparatus that measures surface roughness using an extremely thin diamond needle that scratches the surface of the workpiece is ordinarily used. However, this apparatus requires a workpiece specimen cut from the precisely and mechanically processed surface of an automobile, airplane, ship, etc., to ensure stability on the measurement table. The use of such an apparatus damages the surface of the finished product. If a haptic sensor attached on a bar could be independently used as a part of the surface roughness measurement apparatus, it would protect the product. For example, Shimada devised a haptic sensor attached to the surface of a rubber fingerstall in order to measure surface roughness. The stall could be fitted on a fingertip and then rubbed on the surface of the material being measured [4]. This technique is effective in precision machining.

On the other hand, for skin-roughness measurement in the beauty industry, it is possible to obtain and evaluate, for example, the condition of skin after make-up removal, or the quality of human hair, as measurable numerical data. In addition, the technique is effective for the evaluation of the surface condition of materials such as sharkskin, and is applicable in the field of biotechnology for measuring surface roughness in paper, such as toilet paper, newspaper, and paper diapers [5].

With regard to robots, the inner mechanism of the robot is generally exposed or covered with a skin made of hard material, such as plastic or metal. Even if a rubber material is used, it will not possess haptic sensing like human skin. Therefore, force and thermal sensors must be installed in the material. In the future, it is likely that a skin for use in robots will be developed. Robot skin containing haptic sensors could be used to cover robots like human skin. We may come to have friendly feelings for robots once they begin to play an active part in our lives, as is imminent in terms of helping with household chores and nursing.

Sensors are essential for sensing force and temperature in the inner mechanism of the robot. These correspond to the haptic sensing of human skin. The human skin has five types of touch sensation: tactile, baresthesia, algometry, warm, and cold [6]. At present, robots can sense force and temperature with sensors to represent these five sense perceptions. An independent sensor is used for each. Therefore, many sensors are necessary, and the processing of the information they provide becomes complicated. The development of a simple sensor that handles information for multiple perceptions would be useful. Regarding this requirement, there has been typical and effective investigation of the capability of multi-signal acquisition in one sensor [7]. In addition, sensors are currently vulnerable to the characteristics of their materials, such as tensile and compression strength.

A currently available robot sensor has unique sensitivity to a force applied normal to a touched material. When we need to measure the shear force on the robot, strain gauges or piezoelectric elements must be added [8,9,10,11]. Thus, the sensor is complicated in structure and vulnerable to extrinsic mechanical forces. It therefore lacks extension, and is not suitable for applications where mimicking human skin is required. For a sensor to be useful in the skin of a robot, softness and large extension are important. Furthermore the cost of its production must allow mass production. From these causes, the conception of a skin sensor is very significant, and there are many works on skin sensors, for example: sensing in a human-machine system [12]; piezoresistive sensors utilizing carbon nanotubes [13]; electronic skins (e-skins) [14]. On the other hand, Shimada attempted to develop a sensor that fulfills these demands using natural rubber (NR) mixed with metal particles [15]. Large extension and softness were achieved with NR-latex as the natural rubber. By enhancing the electric conductivity of the natural rubber with metal particles mixed into the NR-latex, haptic sensibility was attained. The metal particles consisted of particles involved in a magnetic compound fluid (MCF) as follows: a magnetic fluid (MF), which is an ordinary intelligent fluid responsive to the magnetic field used in rotating shaft sealing, contains magnetite Fe_3_O_4_ sphere particles on the order of 10 nm. In the initial stage, Shimada devised in 2001 a new intelligent fluid responsive to a magnetic field by compounding MF and metal particles, such as Ni, Fe, Cu, etc., on the order of 1 μm. He named the new fluid magnetic compound fluid (MCF) [16]. Today MCF is defined as a colloidal fluid containing Fe_3_O_4_ and other metal particles on the order of 10 nm dispersed in a solvent. Fe_3_O_4_ particles play a bonding role among the metal particles, allowing numerous metal and Fe_3_O_4_ particles to aggregate. As a result, magnetic clusters formed by the aggregation take shapes such as prolate spheroid, needle, or network, and those shapes can be verified by the new method of extracting magnetic clusters from colloidal fluid devised by Shimada [17]. These magnetic clusters range in size from the order of nm to mm, and are aligned along the magnetic field lines by the application of a magnetic field. MCF is useful for engineering applications owing to the magnetic clusters. It is effective in polishing [18], and as a material for use as a damper [19] and composite material due to these magnetic clusters [20]. As for MCF damper, the damping effect is enhanced by the increasing viscosity of the MCF owing to the magnetic clusters. With regard to MCF polishing, the magnetic clusters act as magnetic brushes, like toothbrushes, and produce an enhanced mirror-finishing effect [21]. Shimada showed that the properties of the fluid in these engineering applications could be enhanced by varying the behavior of the magnetic clusters [22].

The rubber mixed with MCF is called MCF rubber. The magnetic clusters in MCF rubber produce the electric conductivity that leads to sensitivity. At the beginning of MCF rubber research, the rubber was mixed using silicon oil rubber, and vulcanized under a constant temperature and magnetic field application. MCF rubber has a switching effect, which means the electric resistance is large, more than MΩ when touched with almost negligibly small pressure, and this resistance is reduced abruptly up to several Ω by pressure [23]. The electric resistance can be as large as 335 Ω at the split second that an electric current flows, whether the pressure touches the rubber or not; at that time its pressing force F_s_ is 0.294 N. The F_s_ values of the commercial pressure-sensitive electrically conductive rubbers (PSECRs) are larger than this, for example, 6.53 N, 38.1 N and 75.5 N in PSECRs made by the Japanese Co. Ltd. It is generally said that the F_s_ must be on an order less than 0.01 N in order to reflect the sensitivity of human skin.

If natural rubber (NR-latex) is used in place of silicon oil rubber as a constituent of the MCF rubber, the F_s_ becomes less than 0.01 N. In fact, Shimada obtained the result that F_s_ is 0.0149 N [15]. NR-latex constitutes a hydrophilic system, while silicon oil rubber is a lipophilic one. It is easier for an electric current to flow in the former than in the latter. Owing to the higher sensitivity of the sensor made of NR-latex, not only the pressing force but the shear force can be easily measured by the same sensor [15]. In addition, NR-latex is more effective than silicon oil rubber for the sensor requirements of softness and large extension the because of its large elasticity. If a sensor is made of NR-latex, large extension and softness can be achieved.

On the other hand, in a sensor made of PSECR, the shear force cannot be measured easily. The shear force is also known as the friction force; it depends on the normal force, although the normal force is larger. The currently used PSECR sensor is good at sensing the normal force. There is a notion that it might be possible to add to PSECR to measure shear force. However, this idea has the same problems of vulnerability as strain gauges or piezoelectric elements, and would be ineffective, as explained above.

Therefore, the MCF rubber made of NR-latex can simultaneously sense normal, shear forces and temperature, as well as possess large extension and softness. However, the problems of secular changes in the electric resistance [24], higher sensitivity with F_s_ less than 0.01 N, etc. still remain unresolved. The previous method of making the MCF rubber is based on the process by drying the MCF rubber under a constant temperature because of the vulcanization of the rubber as well as by the application of a magnetic field because of the creation of the magnetic clusters in the rubber [15]. By this method its vulcanization becomes unstable, whether the MCF rubber is vulcanized or not. In addition, the characteristics of the MCF rubber undergo secular changes. Therefore, the method used to make the MCF rubber must certainly be investigated.

In the present paper, to fulfill all sensor requirements including haptic sensibility, sensing both shear stress and normal force, sensing temperature, a strong and simple structure, softness, and large extension, we propose a new method of producing sensitive rubber by introducing an electrolytic polymerization method and utilizing NR-latex, MCF, and a magnetic field. We evaluate the effectiveness of the new method by investigating the electric and dynamic characteristics of the rubber. In addition, vulcanization of the MCF rubber by the new method is clarified by analyzing not only its changes of voltage, electric current, and temperature, but also its viscosity using a viscometer. Where we must note that the same force sensor can measure temperature. This is because sensing can be obtained by changes of electric current or voltage, and change in temperature corresponds to that of electric current or voltage. Therefore, we have only to focus on the force apart from the temperature in the case of measuring the property of the rubber in order to investigate the sensitivity of the rubber sensor. In this study, we deal with the electric and dynamic characteristics of the rubber.

## 2. New Method by Electrolytic Polymerization 

**Figure 1:**
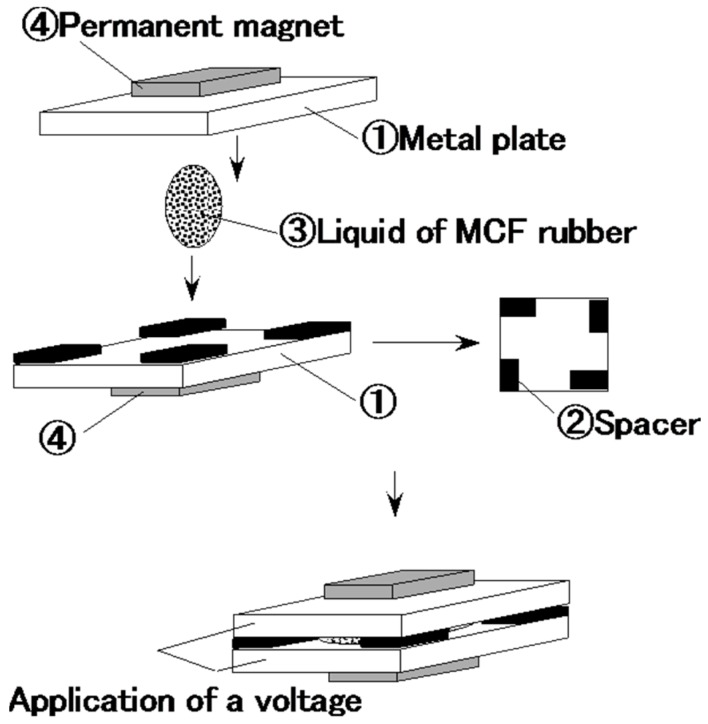
Schematic diagram of new method for making MCF rubber by electrolytic polymerization.

The liquid in the MCF rubber consisted of 12 g Ni powder, with particles on the µm order and pimples on the surface (No. 123 by Yamaishi Co. Ltd., Noda, Japan), 3 g water-based MF with 50 wt.% Fe_3_O_4_ (M-300, Sigma Hi-Chemical Co. Ltd., Tsutsujigasaki, Japan) and 12 g NR-latex (Rejitex Co. Ltd., Atsugi, Japan). Therefore, the mass concentration of the magnetic particles was 48.8 wt.%. These are such optimal combination ratios that allowed us to vulcanize the MCF rubber with 1-mm thickness. MCF rubber liquid was poured on one metal plate and sandwiched by another, as shown in Figure 1. As indicated in the figure, two permanent magnets ④ were applied to the outer side of each metal plate ①. The permanent magnets were rectangles, 10 mm × 15 mm in size, and 5 mm thick. Since the size of the vulcanized MCF rubber depends on that of the permanent magnet, the obtained rubber samples were also 10 mm × 15 mm rectangles. Voltage and current were supplied between the plates ① as indicated in the figure; as may also be seen, the plates were held apart by spacers ② with 1 mm thickness.

The voltage and current were applied for 30 min. This vulcanized the liquid MCF rubber ③. In contrast to this new method, the previous method of making MCF rubber used just a magnetic field.

**Figure 2:**
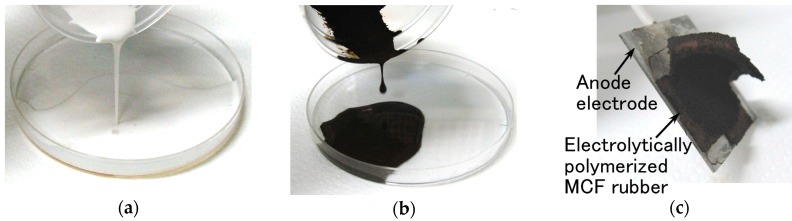
Photographs of our used NR-latex as (**a**), MCF rubber liquid (**b**) before electrolytic polymerization, and vulcanized MCF rubber attached to an anode electrode with a part of removing after electrolytic polymerization (**c**).

Figure 2 shows views of the vulcanized MCF rubber after electrolytic polymerization. It indicates the MCF rubber liquid involving a natural rubber is vulcanized as a solid rubber by electrolytic polymerization.

**Figure 3:**
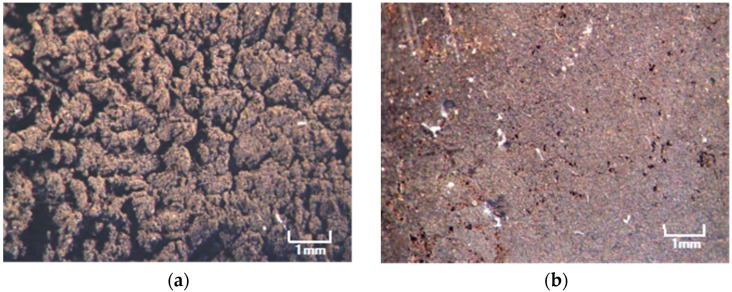
Photographs of the surface at the cathode indicated as (**a**) and anode (**b**) sides of the MCF rubber made by the present new method magnified by a microscope.

**Figure 4:**
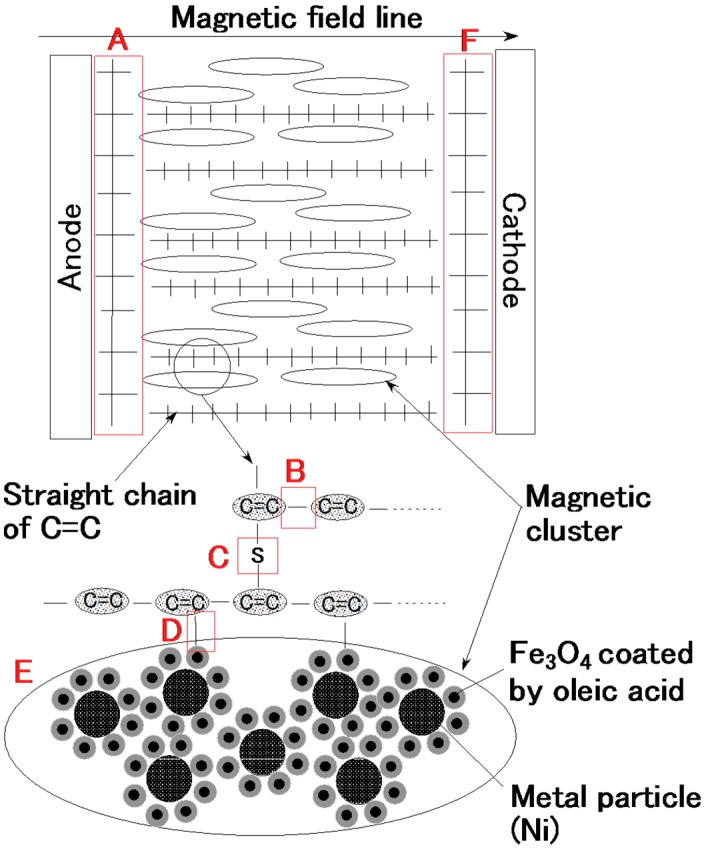
Model of electrolytic polymerization and solidification of the MCF rubber by the present new method.

Figure 3 is a photograph of each surface of the vulcanized MCF rubber at each electrode with the application of 6 V, 2.7 A, 188 mT, the magnetic field strength at the position of the MCF rubber liquid, with a 1 mm space between the electrodes. These voltage, current, the applied voltage and current duration and the magnetic field strength are the optimal conditions so we could vulcanize the MCF rubber with 1 mm thickness. Its surface on the cathode side was rugged, and that on the anode side was smooth. At first, the rubber vulcanized just at the surface of the anode. It then grew incrementally out from the anode surface as shown by Figure 2c. With the application of electricity, the C=C binds of isoprene, a molecule in the NR-latex, were polymerized in a coordinated way, and many C=C bonds were formed in a long chain, as indicated by B in Figure 4. At the same time, the C=C bonds were vulcanized by radical polymerization, as shown by C in Figure 4. In addition, the vulcanization reaction to radical polymerization occurred as a result of the carboxyl group COOH of the oleic acid coating Fe_3_O_4_, bonding C=C and Fe_3_O_4_ as shown by D in Figure 4.

With the application of the magnetic field, the magnetic clusters aligned along the magnetic field lines, which ran in a transverse direction to the two electrodes, as indicated by E in Figure 4. The polymerized C=C were arrayed in the same direction as the magnetic clusters. As a result, vulcanization of the MCF rubber expanded in the direction of the applied magnetic field. At the cathode, the surface of the MCF rubber became rugged, due to the tip of the growing vulcanization. At the anode, cationic polymerization occurred, and at the cathode, anionic polymerization (A and F in Figure 4). When a magnetic field is not applied to the MCF rubber, the polymerized C=C bonds just undergo vulcanization, and the direction of the polymerization is random. Therefore the thickness of the vulcanized MCF rubber and the vulcanization effect are very small.

Electrolytic polymerization focused on the C=C is a well-known procedure in the field of conductive polymer solutions such like a plastic, as in the famous case of polyacetylene polymerized with a Ziegler-Natta catalyst or doping, as demonstrated in the Nobel Prize-winning investigation by Shirakawa, et al. [25]. However, electrolytic polymerization has only been applied to plastics in polymer solutions. It was not applied to rubber until recently, because the effect of electrolytic polymerization on rubber is very small. Still, rubber is one of the components of the polymer solution and it is important to focus on the C=C bonds in natural rubber.

The one of key points on the electrolytic polymerization is the utilization of the C=C bonds involved in the solutions. Therefore, a natural rubber having them can be polymerized radically. These implied viewpoints have been noted in a few other investigations [26,27]. Especially, the report [26] has implied that every rubber having C=C bonds can be subjected to electrolytic polymerization except for natural rubber. However, there have been few investigations utilizing the rubber with C=C bonds. In addition, these investigations did not target on just the electrolytic polymerization of the C=C bonds and the electrolytic polymerization was utilized as an aid for polymerization. These are substantially different from our present investigation in which the focus is on enhancing the electrolytic polymerization of the C=C bonds.

In addition, the effect of electrolytic polymerization can be enhanced by the application of magnetic clusters. Magnetic clusters are so-called fillers that are commonly used in polymer solution. Therefore, both the magnetic field and magnetic filler enhance the effect of electrolytic polymerization. The present study is distinctive in that only a magnetic field was used without the complementary technique of another catalyst or doping in the electrolytic polymerization.

The application of electricity causes electrolysis. The two plates play the role of electrodes. The MCF rubber near the two plates vulcanized into two distinctive materials, shown by A and F in Figure 4, compared to the vulcanized MCF rubber between the plates. The MCF rubber near the anode underwent cationic polymerization (A in Figure 4), and that near the cathode underwent anionic polymerization (F in Figure 4). Therefore, the type of metal plate used is an important factor in determining the component by which type of polymerization occurs. In the present investigation, we used plates made of stainless steel.

**Figure 5:**
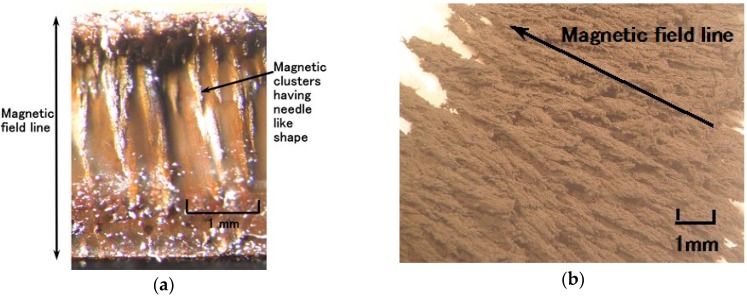
Photograph of cross-section of the MCF rubber made by the present new method magnified by a microscope, shown at (**a**); photograph with microspore of magnetic clusters extracted from the MCF rubber liquid using the magnetic clusters extraction method which was devised by Shimada [17], shown at (**b**).

Figure 5a shows a cross-section of the solidified MCF rubber as observed by microscope. The upper side touches the anode and the lower side the cathode when electricity is applied. The magnetic field is applied transversely to the electrodes. Many magnetic clusters with the appearance of thin needles can be observed. Figure 5b is a microscopic view of the magnetic clusters extracted from the MCF rubber liquid using the extraction method devised by Shimada [17]. The magnetic field was applied from the lower right side diagonally to the upper left side. The magnetic clusters are aligned along the magnetic field lines.

**Figure 6:**
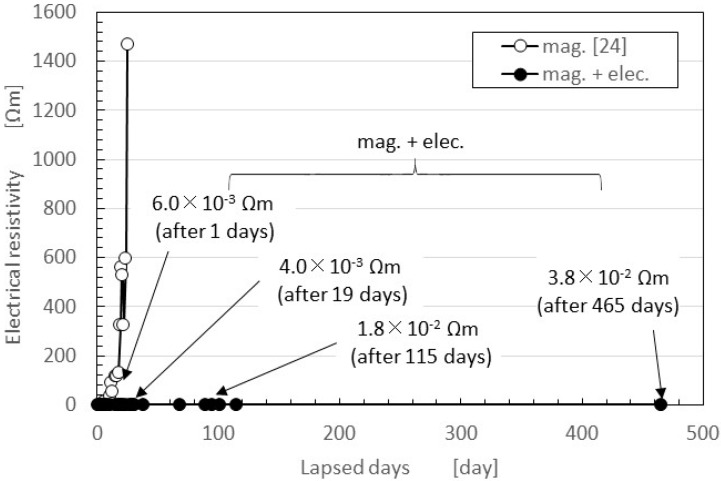
The secular changes in electric resistance between the electrodes opposing each other with no normal force applied to the vulcanized MCF rubber utilizing NR-latex.

The secular changes in the electrical resistivity of the vulcanized MCF rubber created with NR-latex are shown in Figure 6 as volume resistance. In general, the electric property of the material made by the electrolytic polymerization of the conductive polymer solution is ordinarily measured as surface or volume resistance. The latter is suitable for the material with the effect of its thickness. Because the MCF rubber is filled with MCF, the effect of the filler on the electrical property along its thickness direction cannot be neglected. Therefore, in the present study, we adopt the volume resistance.

This figure also presents a comparison between two cases: magnetic field only (i.e., the previous method for making MCF rubber with silicon oil rubber, indicated as “mag.”, that is the same as “mag.” indicated as the following figures, and that means the rubber is vulcanized by drying under a constant temperature 47 °C) [24]; and with both electrolytic polymerization and the magnetic field (i.e., the present new method, indicated as “mag. + elec.”). Although the former case used NR-latex rubber, the period during which the characteristics remained constant was around one week. In contrast, in the latter case, characteristics were stable for more than one year. In addition, in the latter case it was possible to obtain a small electrical resistivity, close to the electrical resistivity of a conductor. Specifically, 3.8 × 10^−2^ Ω·m, as indicated in the figure, corresponds to a surface electric resistance of 2.0 × 10^−6^ Ω/m^2^ as surface resistance, where our NR-latex has ordinarily 10^14^ Ω·m.

The electrical resistivity of conductors is less than 10^−4^ ordered Ω·m. The resistivity of silver and copper, for example, is 10^−7^ ordered Ω·m. Polyphenylene-based conductive plastic doped with ASF_5_ is 10^−2^ ordered Ω·m. On the other hand, the leading PSCER made of natural rubber has the least or smallest degree of 1-ordered Ω·m after thermal degradation. By comparing electrical resistivity after a long lapse of time, we can also obtain a smaller electrical resistivity. The results are dependent on the method used to make MCF rubber using electrolytic polymerization aided by a magnetic field. In the case of mag., the MCF rubber is vulcanized under a constant temperature and application of a magnetic field. The vulcanization of the rubber depends on just the change in the polymerized molecular structure of the rubber induced by thermal energy, and the degree of the vulcanization changes with the atmospheric temperature. On the other hand, in mag. + elec., the vulcanization of the rubber depends on both the electrolytic polymerization of C=C and the magnetic clusters aligned along the magnetic field lines in the same direction as electrolytic polymerization. As shown in the following measurement of the temperature of MCF rubber liquid by electrolytic polymerization, the electric energy converts thermal energy, and the rubber is vulcanized. In this case, we do not need an atmospheric temperature at optimum values to vulcanize the rubber.

## 3. Experimental Procedure

We investigate the properties of MCF rubber produced using the new method of electrolytic polymerization with a magnetic field to check the viability of the method for use in applications. We examine the electric characteristics under normal and shear forces and the dynamic characteristics of tensile rate, which are effective for use in the haptic sensors widely used in various engineering applications.

**Figure 7:**
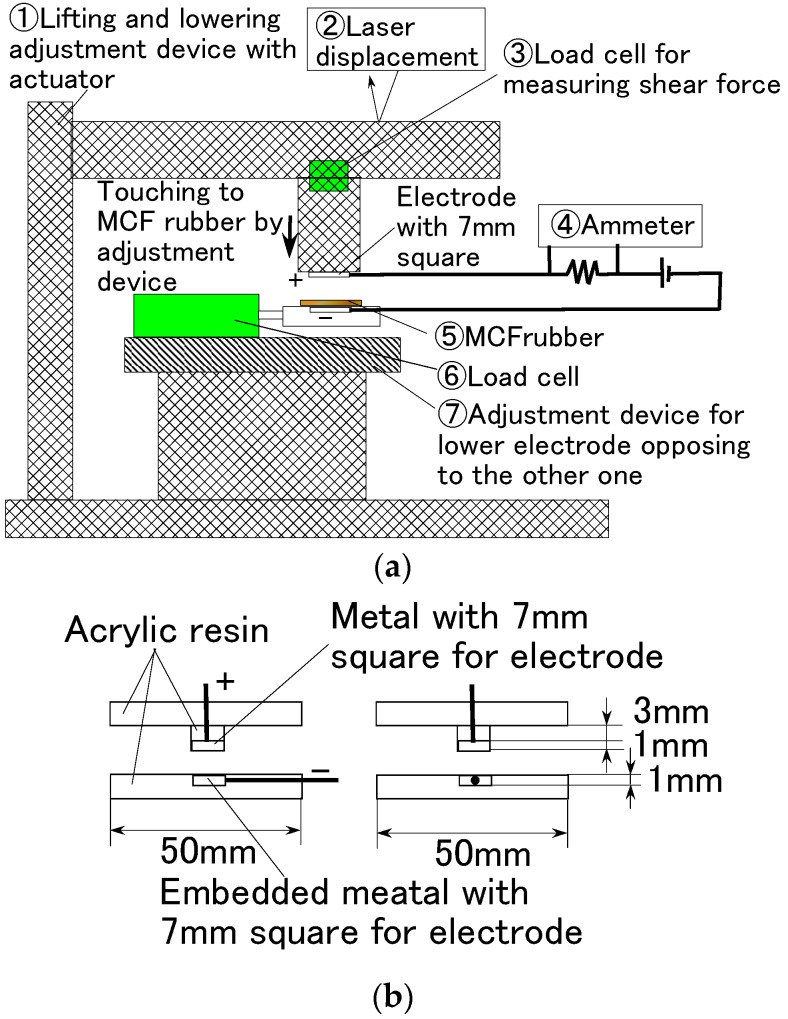
Schematic diagram of experimental apparatus used to investigate the electric characteristics in the case of NFE: (**a**) overview; (**b**) the diagram shows details around the electrodes.

First, we used the experimental apparatus to measure the electric current, voltage, or electric resistance between the opposing electrodes at the application of a normal force to the vulcanized MCF rubber, as shown in Figure 7. This was accomplished using an electric power supply of 10 V and an electric resistance of 1.8 kΩ. The vulcanized MCF rubber was placed between the two electrodes, which were 7 mm stainless steel squares. The upper electrode was moved to touch the lower one by an actuator at a pressing speed of 10 mm/min. The MCF rubber made of silicon oil rubber was 0.5 mm thick, and other MCF test specimens made of NR-latex and PSECR were 1 mm thick. When we consider the actual formation of the MCF rubber as a haptic sensor, the MCF rubber is placed between the counter electrodes. This formation corresponds to the formation of the electrodes in Figure 7b. In addition, the electric resistance conducted by the formation is volume resistance that is suitable for measuring the electric properties of the rubber filled by MCF owing to the effect of the filler. For the sake of simplicity, this experimental procedure will be referred to as the normal force experiment (NFE).

**Figure 8:**
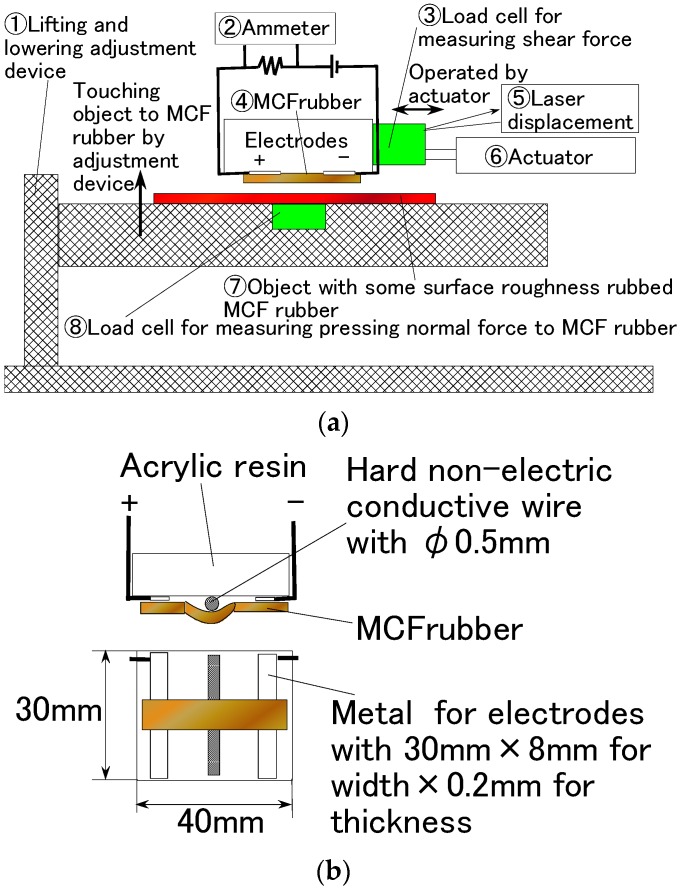
Schematic diagram of experimental apparatus used to investigate the electrical characteristics in the case of SFE: (**a**) overview; (**b**) the diagram shows details around the electrodes.

Second, we used another experimental apparatus to measure the electric current, voltage, or electric resistance within the vulcanized MCF rubber, as shown in Figure 8. Again, the power supply was 10 V and the electric resistance 1.8 kΩ. Both tips of the solidified MCF rubber were placed between the two electrodes of the stainless plate. The MCF rubber touched a rubbing flat plate with a surface roughness of R_a_ = 20.86 μm, R_y_ = 199.9 μm, and R_q_ = 26.89 μm, and was moved parallel to the material surface by an actuator at a speed of 5 mm/s and a sweeping distance of 50 mm. A hard, non-electric body with φ5 mm was interposed between the MCF rubber and the acrylic resin body so that the MCF rubber could be contacted exactly. This touching method was found to be more effective when the contact area was smaller than the MCF rubber as a whole, and with it slightly bent. The bending effect on the MCF rubber has been shown the previous investigation by Shimada [4]. Again, MCF rubber made of silicon oil rubber was 0.5 mm thick, and other MCF test specimens made of NR-latex and PSECR were 1 mm thick. For the sake of simplicity, this experimental procedure is referred to as the shear force experiment (SFE).

Finally, we used a commercial, small-size tensile testing machine (SL-6002, IMADA-SS Co. Ltd., Toyohasi, Japan). All test specimens with rectangular parallelepiped shape were 1 mm thick 10 mm wide, and 10 mm long in the initial stage before tension. The maximum tensile force was 0.5 N, and the speed was 100 mm/min. The tensile stress is repeatedly applied to the rubber five times. Because of the widely known stress softening (Mullins effect), it is investigated in the present rubbers too.

## 4. Electric and Dynamic Characteristics

**Figure 9:**
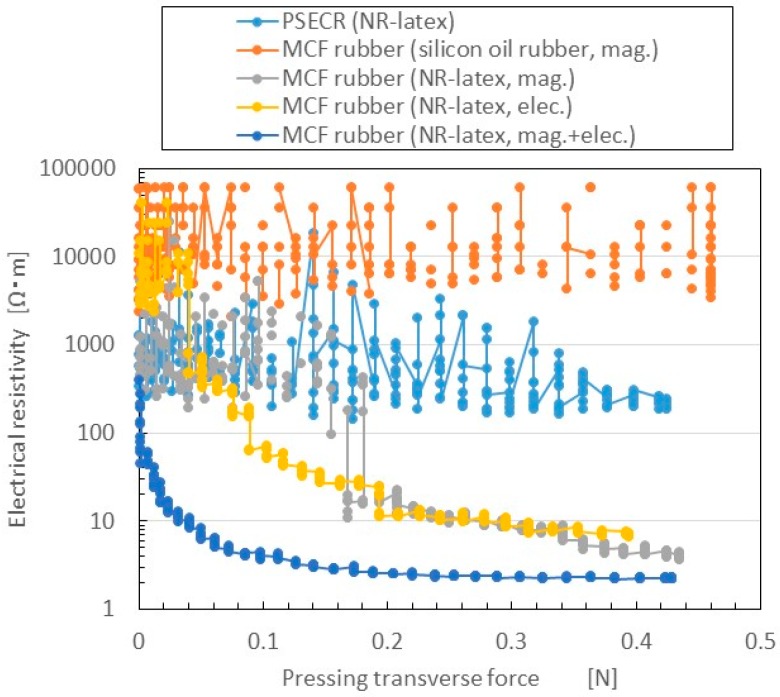
Electric characteristics of MCF rubber made by the new method in the case of NFE, compared among three electric and magnetic field conditions, and among NR-latex, silicon oil, and PSECR rubber.

Changes in electrical resistivity to the pressing transverse force measured by NFE are shown in Figure 9. The figure also presents a comparison of three cases: magnetic field only (mag.); electric field only (i.e., the use of electrolytic polymerization without the magnetic field; elec.); and both electrolytic polymerization and magnetic field (mag. + elec.). The first is the case of MCF rubber by the previous method of making MCF rubber by just drying under a magnetic field application. The third is the case of MCF rubber made by the present new method. The figure also presents the results for PSECR made of NR-latex (PSECR), and MCF rubber made of silicon oil rubber, consisting of 3 g Ni (No. 123), 3 g Cu powder with twig-shaped particles on the order of µm (MF-D2 by Yamaishi Co. Ltd., Noda, Japan), 4 g kerosene-based MF with 50 wt.% Fe_3_O_4_ (HC-50, Taiho Industry Co. Ltd., Takatsuki, Japan), and 10 g silicon oil rubber (SH9550, Shin-Etsu Chemical Co., Ltd, Tokyo, Japan) which is the case of MCF rubber by the previous method vulcanized under a constant temperature and application of a magnetic field (silicon oil rubber, mag.).

**Figure A1:**
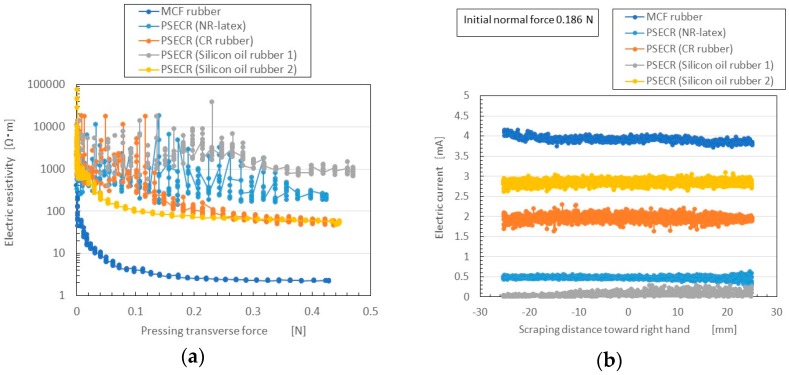
Electric characteristics of other PSECRs compared with that of the MCF rubber made by the present new method: (**a**) in the case of NFE; (**b**) in the case of SFE.

Electrical resistivity in the rubbers made of NR-latex was smaller than in those made of PSECR and silicon oil MCF rubber. In particular, the sensitivity of NR-latex MCF rubber was larger than that of silicon oil MCF rubber, in accord with previous research. The electrical resistivity of elec. and mag. were the largest. As the C=C bonds are polarized, and their electrolytically polarized direction lined up with the direction of the magnetic cluster, more electric current flowed. The rapid decrease in electric resistance in the mag. + elec. was particularly large compared to the other cases. This indicates a larger switching effect, which means that the F_s_ can be as small as possible. F_s_ of MCF rubber (NR-latex, mag. + elec.) is 0.1 mN. This is far above the value that is required in a haptic sensor as described in the previous Introduction section. As previously mentioned, this is important in order to reflect the sensitivity of human skin. As a result, the new method of making MCF rubber with both electrolytic polymerization and a magnetic field is effective for producing a haptic sensor utilizing normal force. Incidentally, other results of the electrical resistivity of PSECR are shown in Figure A1a in the Appendix. The PSECR is less sensitive than MCF rubber made by the new method. Therefore, the MCF rubber made by the new method can be utilized in the various haptic sensing fields as described in the previous Introduction section.

**Figure 10:**
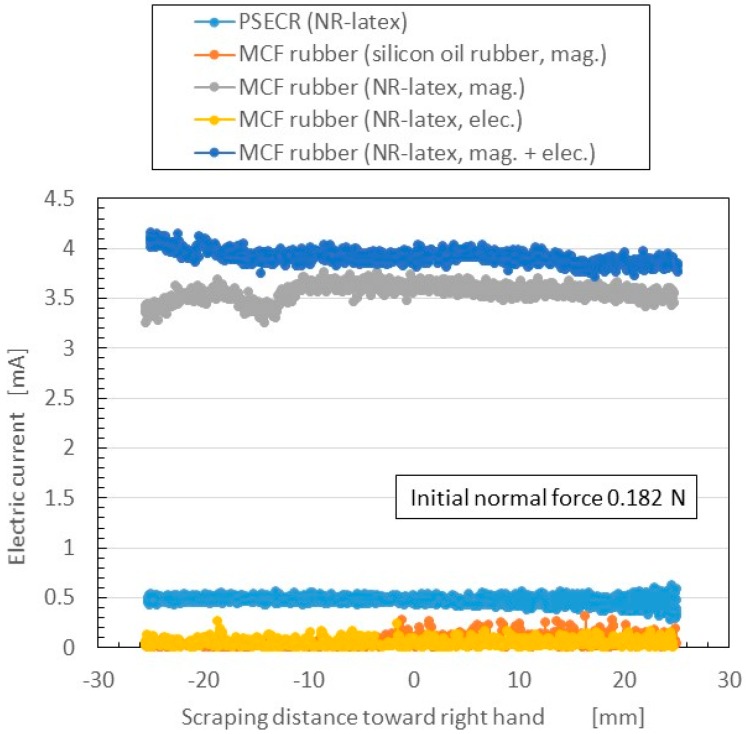
Electrical characteristics of MCF rubber made by the new method in the case of SFE, comparing among three electric and magnetic field condition, and among NR-latex, silicon oil, and PSECR rubber.

The change in electric current flowing inner the rubber to shear force measured by SFE is shown in Figure 10. The normal force changed under shearing motion according to the elasticity of the MCF rubber; the initial normal force before moving is indicated as “Initial normal force”. The figure also presents a comparison of the five cases shown in Figure 9. The more the electric current is, the more sensitive the rubber in the case of utilizing as a sensor with shearing motion. The electric current in rubbers made of NR-latex was larger than that in PSECR and silicon oil MCF rubber. The electric current in electrolytic polymerization without the magnetic field was smaller than that of mag. and mag. + elec. Furthermore, the electric current was largest when both electrolytic polymerization and magnetic field were used. This was because the magnetic clusters and C=C were bonded to one another by the electrolytic polymerization so that the electric current flow was sufficiently complicated to pass reciprocally through both the magnetic cluster and polymerized directions. Thus, the new method of making MCF rubber with both electrolytic polymerization and a magnetic field was effective in producing a haptic sensor utilizing shear force. Incidentally, other results of changes of the electrical current of PSECR are shown in Figure A1b in the Appendix. The PSECR is less sensitive than MCF rubber made by the new method. Therefore, the MCF rubber made by the new method in the case of shearing motion as well as in the case of pressing motion can be utilized in the various haptic sensing fields as described in the Introduction section.

As shown by Figure 6 the endurance period of the MCF rubber made by the new method is very long. The results in Figure 6 were obtained by the same experimental apparatus as that of NFE-type in Figure 9, which has opposite electrodes. Therefore, the repeatability of the measuring normal force produces the same value such that the sensor measuring normal forces are adequate enough to be used as a sensor. The precision of the measuring normal forces in NFE-type and shear stress in SFE-type is less than 10%.

**Figure 11:**
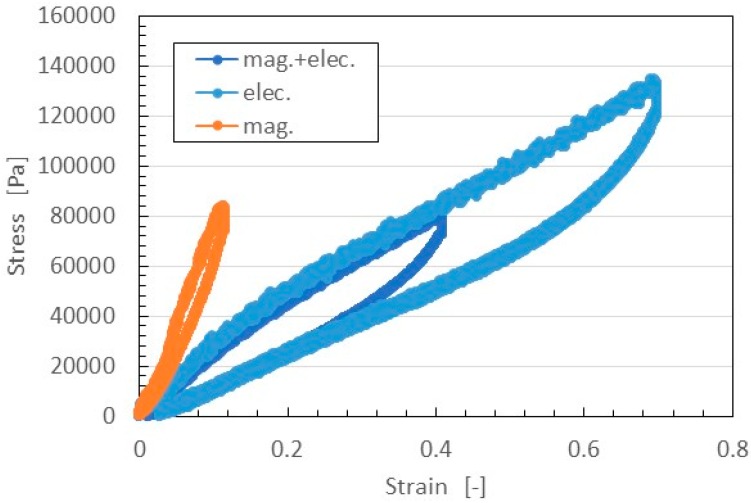
Relation between strain and stress of MCF rubber by tensile testing machine.

The relation between strain and stress is shown in Figure 11. In general, in the case of filled rubber, the stress-strain responses have a well-known hysteresis curve and stress softening (Mullins effect) [28,29]. According to the effect, the stress-strain curve at the first time is different from that after the first, and the stress-strain curves after the first are almost the same as far as the maximum tensile force is the same. Therefore, the figure shows the results of the second application of tensile stress under a maximum tensile force at 0.5 N. The MCF rubber made with the magnetic field only was harder than that made by the application of magnetic and electric fields. This was because, with the former MCF rubber, the magnetic clusters were easily separable from the NR-latex, leaving the elasticity of the rubber dependent on just the vulcanization of the NR-latex itself. In contrast, with the latter MCF rubber, the magnetic clusters were bonded to the NR-latex by the radical polymerization of COOH of Fe_3_O_4_ particles, as shown in Figure 4, The elasticity of the MCF rubber thus depended on both the NR-latex and magnetic clusters.

On the other hand, the MCF rubber made with the electric field only could be easily extended. In addition, the Young’s modulus of that MCF rubber was at the same level as that of the MCF rubber made by the application of both magnetic and electric fields. This was because MCF rubber is easily extended due to the electrolytic polymerization of NR-latex. By compounding Ni and MF, however, this ability to extend is decreased due to the bonding of magnetic clusters to the NR-latex.

## 5. Detailed Mechanism of Solidification of MCF Rubber

**Figure 12:**
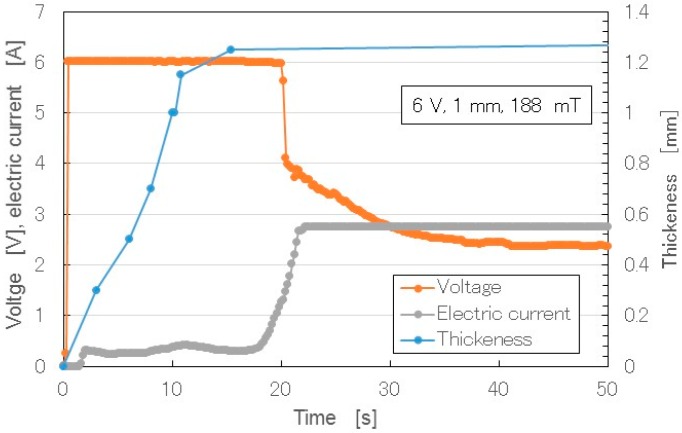
Changes in voltage, electric current, and thickness of MCF rubber over time during the period of vulcanization with the new method.

In the production of MCF rubber, the application of electricity corresponds to an electrolysis process. We measured the electric current and voltage between the electrodes under both electrolytic polymerization and the magnetic field. Figure 12 shows the changes over time of the thickness of the solidified MCF rubber, as well as the electric current and voltage when electricity was applied at 6 V, 2.7 A, and 188 mT, with a 1.3 mm space between the electrodes.

When the electricity was first applied, the voltage was held constant at 6 V, and the electric current was zero because the MCF rubber had not yet vulcanized, and the electric resistance of liquid MCF rubber is large. Soon after the initiation of electricity, the MCF rubber began to polymerize electrolytically and thermally, and thickness increased. In the electrolysis procedure of making MCF rubber, the MCF rubber liquid corresponds to the electrolyte. The electric energy is converted to thermal energy. The thermal energy plays a role in the solidification of the rubber. Therefore, the procedure for making MCF rubber in this study did not require heat drying. When the whole area between the electrodes was completely polymerized electrolytically and thermally, and the metamorphosed structure of the MCF rubber (Figure 4) had settled down, the thickness of the MCF rubber became saturated, and the electric resistance reached a constant value. Therefore, the electric current also increased and became constant. At that time, the voltage decreased to hold constant. In the results shown in Figure 12, there was a 1 mm space between the electrodes. If we had used a larger space, thicker MCF rubber would have been vulcanized. Such vulcanized MCF rubber can be obtained in less than 20 s under the present experimental conditions, making it a very cost-effective process, whereas the thickness of NR-latex electrolytically polymerized by just an electric field is about 0.15 mm. The thickness is the same as that of membrane vulcanized by just drying under atmospheric temperature. Therefore, the applications of the magnetic field and MCF as filler are necessary to enhance the rubber thickness.

**Figure 13:**
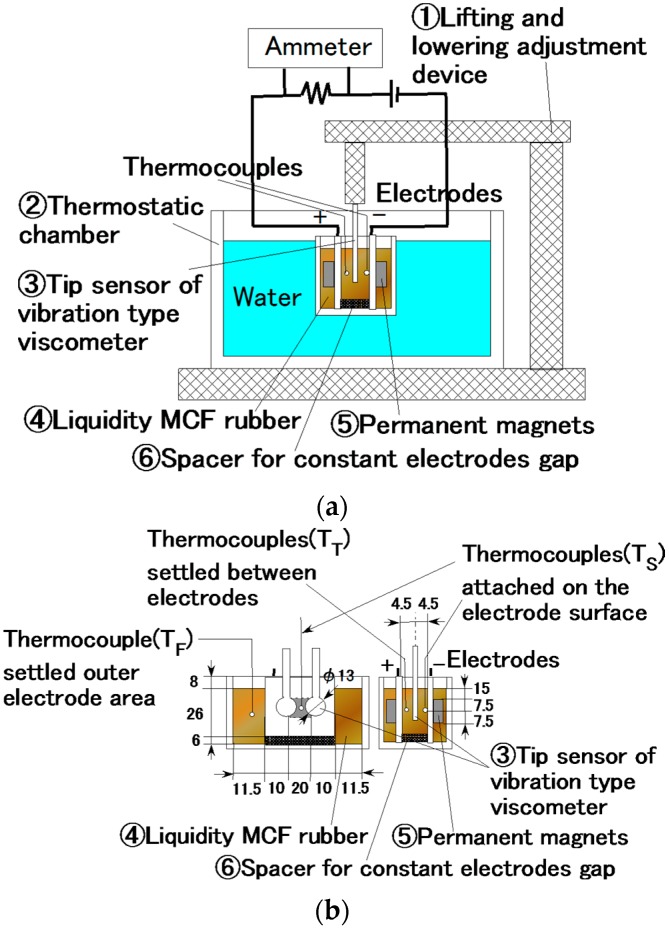
(**a**) Schematic diagram of experimental apparatus used to investigate the vulcanization of MCF rubber by the new method; (**b**) shows the details of the container with MCF rubber liquid, electrodes, and magnets.

By investigating the behavior of the liquid MCF rubber between the electrodes under the application of electricity, the vulcanization process could be clarified in detail. We measured the temperature and viscosity of the MCF rubber between the electrodes under the application of electricity with a magnetic field. As shown in Figure 13, liquid MCF rubber ④ was poured into a container ② dipped in water held at a constant temperature of 20 °C, adjusted by a thermostat, and an electrode with a permanent magnet ⑤ was inserted into the liquid. A vibration-type viscometer tip sensor ③ (a thin plate made of non-conductive material with a diameter of φ13 mm, and 4.5 mm electrode) was inserted between the electrodes, and viscosity was measured under the application of electricity and the magnetic field. The temperature was measured by thermocouples in the rubber liquid at three positions: outside the electrodes, on the surface of the electrode of the cathode within the area of the electric and magnetic fields, and in the area of the electric and magnetic fields between the electrodes. The first position represented the temperature of the MCF rubber liquid unaffected by the electric and magnetic fields; this temperature is referred to as T_F_. The second is the temperature of the liquid influenced immediately by the electric and magnetic fields, T_S_. The third is the temperature of the liquid influenced by the electric and magnetic fields, but not directly, T_T_.

As the effects of both the electrolytic polymerization and magnetic field were large, the viscosity of the solidifying MCF rubber liquid was too large to measure using a vibration-type viscometer because it was outside the range of the instrument. Therefore, the mass concentration of the magnetic particles of Fe_3_O_4_ and Ni was adjusted to 33.8 wt.%. Although this is smaller than in the preceding experiment (48.8 wt.%), the qualitative tendencies of the thickness of the solidifying MCF rubber were the same.

Because there was not much distance between the inserted tip sensor of the vibration-type viscometer and the plate (see Figure 13), we had to take the scale effect into account. For example, when the viscosity of the MCF rubber liquid was 12000 mPa·s, it had a scale effect of 19% owing to the small gap of electrodes. The viscosity shown in Figure 13 was the obtained viscosity minus the viscosity that resulted from the scale effect.

**Figure 14:**
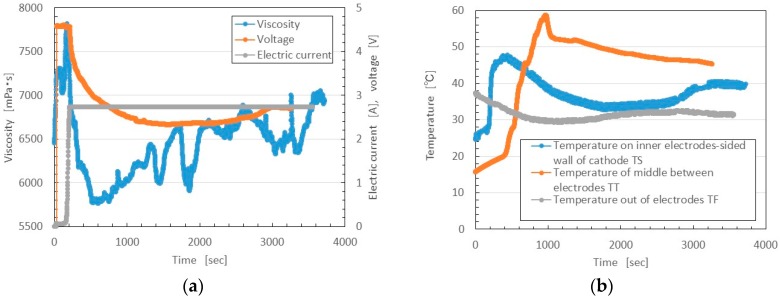
Changes in viscosity, electric current, voltage, and temperature of MCF rubber liquid over time during the vulcanization by the new method: (**a**) viscosity, voltage and electric current; (**b**) T_S_, T_T_ and T_F_.

During the initial vulcanization, the voltage or electric current time is short, as shown in Figure 12. In the present experiment, however, the volume of the MCF rubber liquid poured into a container was more than that in the case shown in Figure 12. Therefore, the period of initial vulcanization before the voltage began to decrease and the electric current to become saturated was longer, about 200 s, as shown in Figure 14. At this initial vulcanization period, the MCF rubber liquid was vulcanized prominently near the electrodes, and its viscosity then increased until it reached a maximum, together with T_S_. After that, the vulcanization spread over the whole area between the electrodes, viscosity increased gradually, and the value of T_T_ reached its maximum.

## 6. Conclusions

In one experiment investigating the electrical sensitivity with the application of normal and shear force to MCF rubber, as well as the dynamic quantities with a tensile testing machine, we clarified the correlation between electrolytic polymerization and the magnetic field. In another experiment investigating the viscosity and temperature of MCF rubber liquid in the process of vulcanization under electric and magnetic fields, vulcanization performance was clarified. 

By applying electrolytic polymerization focused on the C=C bonds in natural rubber to NR-latex, and also applying a magnetic field and magnetically responsive fluid such as MCF, it is possible to increase the effect of electrolytic polymerization in NR-latex like in plastic in polymer solutions, and to vulcanize MCF rubber quickly. By utilizing a magnetic field, we controlled the magnetic clusters that serve as filler in the polymer solution and align with the electrolytically polymerized C=C bonds along the magnetic field line. This new method of MCF rubber vulcanization is distinctive in that it uses just a magnetic field without another catalyst or doping in the electrolytic polymerization. We enhanced the electric conductivity as well as the vulcanization. Regarding the former, we also enhanced its sensitivity to normal and shear forces exerted on the MCF rubber. In addition, we enhanced the elasticity compared to application of just the magnetic field. The present method has the capability to be applied to other kinds of rubber having isoprene C=C bonds.

The application of electricity corresponds to an electrolysis, therefore, the type of metal plate used is an important factor. In addition, the applied electric conditions of the voltage, current, and the magnetic field strength are also important. The vulcanization of the MCF rubber varies in correlation with these factors. These variations would be explored in future research to be described in our next report. However, the factors shown in the present paper are suitable for the vulcanization of MCF rubber in a short time, making the present method effective for use in haptic sensors in various engineering applications.

## Appendix A

Figure A1 shows the electric resistivity of some PSECRs made of NR-latex (NR-latex), CR rubber (CR rubber) and silicon oil rubber (Silicon oil rubber 1, 2), compared with that of MCF rubber made by the present new method (MCF rubber) in Figure A1a for the case of NFE, and the electric current in Figure A1b for the case of SFE. The electric resistivity of the all PSECRs is larger than the MCF rubber and the electric current of the all PSECRs is smaller than the MCF rubber.
